# Wettability Effect on Nanoconfined Water’s Spontaneous Imbibition: Interfacial Molecule–Surface Action Mechanism Based on the Integration of Profession and Innovation

**DOI:** 10.3390/nano15181447

**Published:** 2025-09-19

**Authors:** Yanglu Wan, Wei Lu, Yang Jiao, Fulong Li, Mingfang Zhan, Zichen Wang, Zheng Sun

**Affiliations:** 1 School of Innovation and Entrepreneurship, Wuchang University of Technology, Wuhan 430200, China; 2315363151@wtu.edu.cn (Y.W.); jiaoyang@wut.edu.cn (Y.J.); lifulong@wut.edu.cn (F.L.); jwc@wut.edu.cn (M.Z.); wangzichen@wut.edu.cn (Z.W.); 2 Hubei Engineering Research Center for BDS-Cloud High-Precision Deformation Monitoring, Wuhan 430223, China; 3 School of Education, Huazhong University of Science and Technology, Wuhan 430076, China; 4 State Key Laboratory of Fine Exploration and Intelligent Development of Coal Resources, China University of Mining and Technology, Xuzhou 221116, China

**Keywords:** Wettability effect, spontaneous imbibition, enhanced viscosity, water slip, analytical model

## Abstract

The effect of molecule–surface interaction strength on water becomes pronounced when pore size shrinks to the nanoscale, leading to the spatially varying viscosity and water slip phenomena that break the theoretical basis of the classic Lucas–Washburn (L-W) equation for the spontaneous imbibition of water. With the purpose of fulfilling the knowledge gap, the viscosity of nanoconfined water is investigated in relation to surface contact angle, a critical parameter manifesting microscopic molecule–surface interaction strength. Then, the water slip length at the nanoscale is determined in accordance with the mechanical balance of the first-layer water molecules, which enlarges gradually with increasing contact angle, indicating a weaker surface–molecule interaction. After that, a novel model for the spontaneous imbibition of nanoconfined water incorporating spatially inhomogeneous water viscosity and water slip is developed for the first time, demonstrating that the conventional model yields overestimations of 16.7–103.2%. Hydrodynamics affected by pore geometry are considered as well. The results indicate the following: (a) Enhanced viscosity resulting from the nanopore surface action reduces the water imbibition distance, the absolute magnitude of which could be 3 times greater than the positive impact of water slip. (b) With increasing pore size, the impact of water slip declines much faster than the enhanced viscosity, leading to the ratio of the nanoconfined water imbibition distance to the result of the L-W equation dropping rapidly at first and then approaching unity. (c) Water imbibition performance in slit nanopores is superior to that in nanoscale capillaries, stemming from the fact that the effective water viscosity in nano-capillaries is greater than that in slit nanopores by 5.1–22.1%, suggesting stronger hydrodynamic resistance. This research is able to provide an accurate prediction of spontaneous imbibition of nanoconfined water with microscopic mechanisms well captured, sharing broad application potential in hydraulic fracturing water analysis and water-flooding-enhanced oil/gas recovery.

## 1. Introduction

The spontaneous water imbibition phenomenon can be easily observed at the microscale, where capillary pressure, the pressure difference resulting from the meniscus between the water phase and adjacent phase, becomes comparable to the weight of the confined water [1,2]. To capture the critical mechanical balance that promotes the movement of confined water, the classic Lucas–Washburn (L-W) equation [3,4] was developed by coupling gravity, capillary force, and viscous force. Based on this profound framework, massive insightful improvements have been made over decades to account for the effects of pore shape, fractal characteristics, and others. However, the prediction accuracy of the classic model encounters a great reduction when pore size shrinks from the microscale to nanoscale, confirmed by lots of molecular dynamics simulations and experimental data [5,6,7]. A clear understanding of nanoscale spontaneous water imbibition dynamics has broad application scenarios, such as evaluation of the formation damage caused by fracturing fluid invading shale or coal seams with rich nanopores, primary gas–water distribution features in unconventional gas reservoirs [8,9], purification of seawater [10], and so on. Hence, it is urgent to shed light on spontaneous water imbibition behavior at the nanoscale.

The essential mechanisms of spatially varying viscosity and water slip at the wall surface, which take effect at the nanoscale, are responsible for the invalidity of the L-W equation. As reported, the viscosity of water with a distance of only several molecular diameters from the pore surface differs greatly from bulk water [11,12]. In contrast, the viscosity of water in the middle region of the nanopore behaves the same as bulk water. As a result, water viscosity at the nanoscale shows a dependence on water–surface distance, completely different from the macroscale. Inherently, the molecule–surface interaction is the main cause triggering unusual viscosity characteristics, which become much stronger at the nanoscale than at the macroscale. Affected by the surface-induced attraction, water molecules near the nanopore surface align in an orderly fashion, fundamentally changing the water viscosity [13,14,15]. Meanwhile, water molecules away from the action distance of the nanopore surface molecules can move in a disorderly fashion, just like water at the macroscale. Moreover, since surface wettability is the core attribute affecting the surface–molecule strength, the spatially varying feature of nanoconfined water viscosity would be a function of surface wettability, which however has received little research attention. In the other aspect, water molecules at the nanopore surface gain the ability to move, which is termed the water slip phenomenon [16,17,18], another essential mechanism complicating spontaneous water imbibition at the nanoscale. The flow flux of nanoconfined water could be several orders of magnitude faster than that predicted by the classic Hagen–Poiseuille (H-P) equation, demonstrated by high-precision experiments [19,20]. It is still challenging to reveal the exact mechanism behind nanoconfined water slip behavior, but tremendous research efforts have demonstrated that water slip is related to pore size, surface wettability, surface roughness, and so on. In short, the critical issue in investigating spontaneous imbibition of nanoconfined water is to accurately characterize the spatially varying water viscosity and water slip behavior at the nanoscale. As it is almost impossible to incorporate all the possible influential factors in a single technical study, the wettability effect, affecting both nanoconfined water viscosity and water slip simultaneously, is the main focus herein.

Attracted by its broad application potential, a lot of research approaches have attempted to investigate the spontaneous imbibition of nanoconfined water. Molecular dynamics (MD) simulation is widely acknowledged as the most appropriate tool to explore molecular behavior at the nanoscale [21,22], as it is capable of simulating the movement of each particle considering both intermolecular and molecule–surface interactions over extremely small timespans, like picoseconds or femtoseconds. Considering that a basic system for nanoconfined water behavior consists of thousands of individual particles, predicting spontaneous water imbibition utilizing MD will inevitably be computationally costly and time-consuming. At the same time, the reliability of the MD results depends heavily on whether the selected force-field parameters could achieve an excellent match with the simulated physical water–solid properties [23,24], which yields uncertainty as well. More importantly, a separate MD simulation can only examine the effect of a specified solid phase on nanoconfined water flow behavior; it has difficulty elaborating on the wettability effect, which can be regarded as the gradually varying molecule–surface strength, on spontaneous water imbibition performance. Similarly, advanced experimental devices, such as CT, NMR, etc., are utilized to measure water imbibition with different operation conditions [25,26], but they still have challenges in investigating the wettability effect on nanoconfined water imbibition, with each additional experiment requiring a separate experimental setup. Therefore, although numerous researchers using MD or experiments have worked on the spontaneous imbibition of nanoconfined water, the majority of them have focused on the flow dynamics with a relatively fixed solid-phase property. The varying wettability effect on spontaneous water imbibition is still an unsolved knowledge gap.

In contrast, an analytical or semi-analytical model established on reasonable assumptions could yield instantaneous results while maintaining relatively high precision, which is more suitable for investigating the effect of molecule–surface strength on spontaneous water imbibition. Based on the fundamental framework proposed by the L-W equation, a great deal of improvements have been made to extend its applicability from the microscale to the nanoscale [27,28,29]. The slip length, defined as the ratio of the water slip velocity on the surface to the shear rate, representing the magnitude of water slip, has been widely incorporated recently [30,31,32]. Wang et al. systematically reviewed a recently proposed model for water imbibition at the nanoscale, and concluded that water slip is the key mechanism improving the water imbibition ability and cannot be overlooked [33]. However, the slip length used in Wang’s derived model is a constant value, failing to account for the wettability effect on water slip behavior, just like the majority of existing relevant research. Moreover, few studies have paid attention to the spatially varying water viscosity at the nanoscale, leading to the predicted water imbibition length being longer than expected. Feng et al. developed a model to characterize varying water viscosity inside nanopores by accounting for the effect of molecule–surface interactions on the activation energy barrier [34], while another quasi-universal correlation between slip length and wettability was utilized. Thus, in Feng’s model, the theoretical basis of the spatially varying water viscosity is not the same as that for slip length, which may result in doubtful results. Accordingly, currently proposed models fail to effectively couple the spatially varying water viscosity and slip phenomena simultaneously, resulting in their inability to reproduce water imbibition behavior at the nanoscale.

With the motivation of filling the knowledge gap, a profound framework for spontaneous imbibition of nanoconfined water incorporating spatially varying water viscosity and water slip behavior is established for the first time, where the slip length is derived from the nanoconfined varying viscosity in accordance with the microscopic mechanical balance of the first-layer water molecules near the surface. At first, a practical correlation between water viscosity and surface wettability, characterized by contact angle, is utilized here, which has been well validated by massive collected experimental data and MD simulation results [35,36]. Then, the key slip length is derived and is related to surface contact angle, sharing the same theoretical basis as the spatially varying viscosity. After that, a novel model for spontaneous water imbibition considering wettability effects is proposed, which can be further improved by coupling gravity, pore tortuosity, osmotic pressure, etc., to extend its application.

The paper is organized as follows. Basic physical mechanisms underlying the spontaneous imbibition of nanoconfined water are presented first, including enhanced water viscosity while approaching the nanopore surface and the water slip phenomenon. Subsequently, a mathematical model for spontaneous water imbibition at the nanoscale is derived and verified against the existing models and collected MD simulation results. After that, the impact of surface wettability and pore shapes, encompassing nano-capillaries and nano-slits, on nanoconfined water imbibition behavior is revealed. Finally, several conclusions are drawn.

## 2. Physical Model

**Figure 1:**
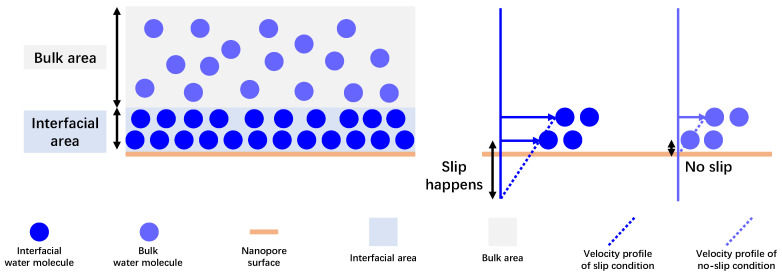
Spatially varying water viscosity and water slip behavior at nanoscale.

It is important to gain a clear understanding of physical mechanisms that result in the mysterious spontaneous water imbibition behavior at the nanoscale, which are spatially varying water viscosity and water slip in this work. The primary cause triggering spontaneous water flow is positive capillary pressure, which implies the presence of hydrophilic surface wettability and that the water–surface contact angle in this investigation is less than 90°. As displayed in Figure 1, it can be observed that water molecules in the interfacial area align in an orderly fashion, while water molecules behave the same as the disorderly molecular thermodynamics in the bulk area. In accordance with the physical meaning of fluid viscosity, representing the magnitude of intermolecular internal friction force during fluid movement, the viscosity of orderly aligned water is larger than that of bulk water. Theoretically, with increasing distance from water molecules to the nanopore surface, water viscosity should gradually decline and approach the bulk water viscosity. For simplicity in developing an analytical model, the whole area of the nanopore is divided into the interfacial area, where water molecules fall within the action distance of the nanopore surface and have greater viscosity, and the bulk area, where water molecules are free of surface–molecule interactions. Another mechanism investigated is water slip at the nanopore surface; in contrast, the water molecules near the surface are generally believed to lack mobility at the macroscopic scale [37,38]. The slip length, presented in Figure 1, is commonly utilized to quantify how fast the near-surface water molecule can move, which can exceed several orders of nanometers, explaining the extremely rapid flow efficiency in nanopores as previously reported [39,40]. By experimental observations and MD simulations, slip length is mainly subject to pore size and surface wettability.

In essence, both the spatially varying viscosity and water slip phenomena are caused by strong molecule–surface interaction strength, which can be reasonably neglected at the microscale or macroscale. Hence, with the intent of establishing a profound theoretical model, the formulas for both mechanisms that share the same underlying cause should be derived consistently, instead of from two different sources. To maintain the consistency, the slip length is derived from the wettability-related viscosity based on the mechanical balance of the first-layer water molecules near the surface, which is the primary difference compared with the other few documents that cover both mechanisms.

## 3. Model Establishment

In accordance with the basic framework of the L-W equation, a novel model for the spontaneous imbibition of nanoconfined water incorporating spatially varying water viscosity and slip length is established. Since nanoconfined water viscosity and the slip length are related to the water–surface contact angle, a key parameter characterizing the surface wettability, the established model is able to reveal the wettability effect on water filling behavior at the nanoscale. The frequently observed nanopore shapes, including nano-capillaries and nano-slits, are considered as well.

### 3.1. Nanoconfined Water Viscosity

A few molecular simulation results indicate that the viscosity of water in the interfacial area is different from that of bulk water and can be characterized as a function of surface wettability. As for the thickness of the interfacial area, representing the effective distance to which the solid nanopore surface could impose an action force on water molecules, previous research efforts proved that the thickness of 0.7 nm could reach excellent agreement with experimental findings and MD results. A simple yet practical model relating nanoconfined water viscosity with surface wettability is utilized here [41]. As demonstrated by the correlation, the water viscosity in the interfacial area could be 3 times that of bulk water with an extremely strong hydrophilic nanopore surface [42,43]. Conversely, as for the hydrophobic wettability with a surface contact angle over 150°, water viscosity in the interfacial area is less than that of bulk water, suggesting that the hydrophobic surface is capable of contributing to water flow efficiency.

(1) $$\frac{\mu_{i}}{\mu_{b}}=-0.018\theta +3.25$$

where *μ_i_* represents the water viscosity in the interfacial area, cp; *μ_b_* represents the bulk water viscosity, which is not affected by the surface–water interactions, cp; *θ* represents the surface contact angle in degrees.

According to the volume-average method, the effective water viscosity in the nanopore considering the difference in the spatially varying water viscosity is as follows:

(2) $$\mu_{eff}=\mu_{i}\frac{A_{i}}{A_{t}}+\mu_{b}[1-\frac{A_{i}}{A_{t}}]$$

where *μ_eff_* represents the effective water viscosity in the whole nanopore space; *A_i_* represents the cross-sectional area of the interfacial area, m^2^; *A_t_* represents the total cross-sectional area of the nanopore, m^2^.

The calculation formulas for the ratio of *A_i_* to *A_t_* in a nano-capillary or nano-slit are below, confirming that the effective nanoconfined water viscosity approaches the bulk water viscosity while pore size becomes much greater than the thickness of the interfacial area. Also, the nanoconfined water viscosity deviates more than the bulk water viscosity when pore size becomes comparable to the thickness of the interfacial area, implying the intensifying nanoconfinement effect at the nanoscale.

(3) $${\left.\frac{A_{i}}{A_{t}}\right|}_{cap}=1-{\left(1-\frac{h}{R}\right)}^{2}$$

(4) $${\left.\frac{A_{i}}{A_{t}}\right|}_{slit}=\frac{2h}{H}$$

where *h* is the thickness of the interfacial area, which is 0.7 nm in this research [44,45]; *R* is the radius of the nano-capillary, nm; *H* is the width of the nano-slit, nm.

### 3.2. Water Slip Length

Another underlying mechanism leading to anomalous spontaneous water imbibition at the nanoscale is the water slip, indicating that the nanoconfined water molecules, particularly the water molecules at the surface, could move faster than expected regularly. For simplicity, the slip length, a physical parameter capturing the movability of the first-layer fluid molecules next to the nanopore surface, is widely utilized. And the slip velocity can be described as a function of slip length and the velocity gradient of the first-layer water molecules in the direction normal to their flow.

(5) $$v_{st}=l_{st}\frac{\partial v_{st}}{\partial z}$$

where *v_st_* is the slip velocity at the boundary, m/s; *l_st_* is the slip length, nm; *z* represents the distance in the direction normal to the fluid flow, m.

Notably, the rationality of Equation (5) stems from the “continuum flow” assumption [46,47], assuming the distance between the first-layer water molecules and pore surface is negligible. However, the distance, commonly believed to be equal to the diameter of a fluid molecule, becomes comparable to the pore size at the nanoscale and therefore cannot be overlooked. Thus, characterizing the flow velocity of the first-layer water molecules is critical for nanoconfined water behavior, rather than the flow velocity at the boundary as Equation (5) describes. Furthermore, according to the definition of slip length, the velocity of the first-layer water molecules adjacent to the nanopore surface is below:

(6) $$v_{s}=l_{s}\frac{\partial v_{s}}{\partial z}$$

where *v_s_* is the velocity of the first-layer water molecules, m/s; *l_s_* is the effective slip length, nm.

Supposing the water flow in a pore where the spatially varying water viscosity is neglected, the viscous force resulting from the water flow at the boundary has the following expression:

(7) $$F=\mu_{b}S\frac{\partial v_{b}}{\partial z}$$

where *F* is the viscous force, N; *S* is the cross-sectional area for the water flow, m^2^; *v_b_* is the velocity of the first-layer water molecules.

Supposing the water flow in a pore considering the presence of a nanoconfined interfacial area, similarly to the derivation process of Equation (7), the viscous force for nanoconfined water is below:

(8) $$F=\mu_{i}S\frac{\partial v_{i}}{\partial z}$$

where *v_i_* is the velocity of the first-layer water molecules considering the change in water viscosity in the interfacial area, m/s.

Combining Equation (7) with Equation (8), the following expression can be derived:

(9) $$\frac{\partial v_{i}}{\partial z}=\frac{\mu_{b}}{\mu_{i}}\frac{\partial v_{b}}{\partial z}$$

In accordance with Equation (6) and the correlation between velocity gradient and slip length, the following expression can be further derived:

(10) $$\frac{v_{s}}{l_{s,i}}=\frac{\mu_{b}}{\mu_{i}}\frac{v_{s}}{l_{s,b}}$$

where *l_s,i_* is the slip length for water flow considering the spatially varying water viscosity, nm; *l_s,b_* is the slip length for water flow neglecting the spatially varying water viscosity, nm.

After further organization, we can obtain

(11) $$l_{s,i}=l_{s,b}\frac{\mu_{b}}{\mu_{i}}$$

As explained, since the slip phenomenon and spatially varying water viscosity share the same origin to assure theoretical consistency in this research, the slip phenomenon for water flow neglecting the presence of interfacial area disappears; therefore, the slip velocity at the boundary is zero and the slip length is equal to the molecular diameter.

(12) $$l_{s,b}=\sigma$$

where *σ* is the water molecular diameter, which is 0.4 nm.

By substituting Equations (1) and (12) into Equation (11), the slip length for nanoconfined water can be derived:

(13) $$l_{s,i}=\frac{\sigma }{-0.018\theta +3.25}$$

In accordance with Equation (13), the water slip phenomenon is related to surface wettability, which becomes pronounced with increasing surface contact angle. As for hydrophobic surfaces, the slip length could be relatively large, which would greatly promote the water flow efficiency at the nanoscale, having an excellent match with the obtained MD results and experimental observations that the nanoconfined water flow capacity surpasses that evaluated by the conventional Navier–Stokes (N-S) equation [48,49,50]. Meanwhile, the slip length declines dramatically with reducing surface contact angle, demonstrating that the strong attraction force exerted by the nanopore surface could effectively reduce molecular mobility at the boundary.

### 3.3. Nanoconfined Capillary Filling

The spatially varying viscosity and slip phenomena, which have an increasingly critical role in affecting water behavior at the nanoscale, are both well coupled in the model for the spontaneous imbibition of nanoconfined water. As for spontaneous imbibition dynamics, the primary force that drives the water to move forward is the capillary force, contributed by surface–water interface tension. In accordance with the balance between capillary force and viscous force, the average velocity for water imbibition is below:

(14) $$V_{ave,cap}=\frac{2\delta \cos\theta }{R}\frac{R^{2}+4l_{s,i}R}{8\mu_{eff}L}$$

where *V_ave,cap_* is the average velocity of water imbibition inside a nanoscale capillary, m/s; *L* is the water imbibition distance, m.

The average velocity can be described as follows as well:

(15) $$V_{ave,cap}=\frac{dL}{dt}$$

Substituting Equation (15) into Equation (14), the analytical model for spontaneous water imbibition in a nanoscale capillary can be derived:

(16) $$L_{cap}=\sqrt{\frac{\delta \cos\theta }{2\mu_{eff}}(R+4l_{s,i})t}$$

where *L_cap_* is the water imbibition distance inside a nanoscale capillary with pore radius of *R*, m; *t* is the timespan for spontaneous water imbibition, s.

Regarding Equation (16), after neglecting the spatially varying viscosity and water slip at the boundary, it degenerates to the classic L-W equation for cylindrical pores, demonstrating the validity of the derived model to a certain extent.

(17) $$L_{cap,ori}=\sqrt{\frac{\delta R\cos\theta }{2\mu_{b}}t}$$

where *L_cap,ori_* is the imbibition distance evaluated by the classic L-W equation for the cylinders, m.

**Figure 2:**
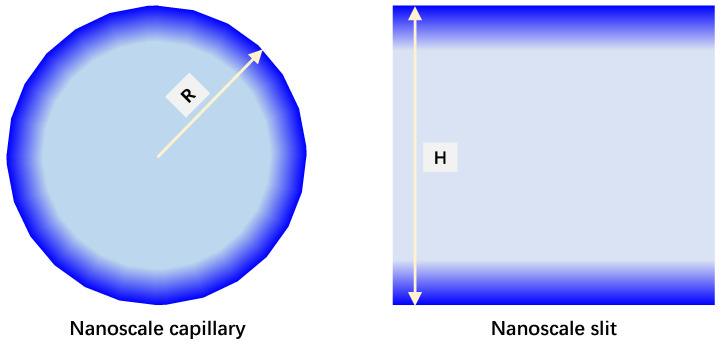
Spatially varying water viscosity feature at nanoscale with different pore shapes.

Similarly, the analytical model for nanoconfined water imbibition in slit-shaped pores (Figure 2) is derived as well, facilitating the presentation of the impact of nanopore shapes, which are provided as Equations (18) and (19). Also, Equation (19) can degenerate to Equation (20), the classic L-W equation for the slit-shaped pores, by overlooking the nanoconfined effect.

(18) $$V_{ave,slit}=\frac{2\delta \cos\theta }{H}\frac{H^{2}+6l_{s,i}H}{12\mu L}$$

(19) $$L_{slit}=\sqrt{\frac{\delta \cos\theta }{3\mu_{eff}}(H+6l_{s,i})t}$$

(20) $$L_{slit,ori}=\sqrt{\frac{\delta H\cos\theta }{3\mu_{b}}t}$$

where *V_ave,slit_* is the average velocity of water imbibition inside a nanoscale capillary, m/s; *L_slit_* is the water imbibition distance inside a slit-shaped nanopore with a height of *H*, m; *L_slit,ori_* is the imbibition distance evaluated by the classic L-W equation for the slits, m.

By modifying the water viscosity and incorporating the term of slip length, the novel analytical models, Equation (16) and Equation (19), respectively, for spontaneous imbibition of nanoconfined water in nanoscale capillaries and slits are proposed. The critical novelty of the models mainly stems from the effective nanoconfined water viscosity that is inherently related to surface wettability, and water slip length derived from the spatially varying water viscosity to ensure the theoretical consistency.

## 4. Model Validation

Before investigating spontaneous water imbibition performance at the nanoscale, it is crucial to examine the reliability of the proposed model in this work. MD simulations have a fundamental theoretical basis with repeatedly verified force field parameters, which can yield accurate nanoconfined fluid behavior, particularly fluid dynamics at the nanoscale within extremely small timespans. Hence, MD simulation results, obtained by Stroberg et al., 2012 [51], characterizing water imbibition in a silica nano-capillary with a diameter of 5 nm and a contact angle of 25°, are employed here as the benchmark data. In other words, theoretical models established by Wang and Rahman [52], He [53], and Wang et al. [33], are selected here for detailed comparison, which can represent the most recent updates on the analytical models for spontaneous water imbibition at the nanoscale. Other than capillary force, Wang and Rahman [52] accounted for the displacement pressure, osmotic pressure induced by the ion concentration difference, and the compression of free gas. The model of He [53] considered the water slippage at the nanopore surface, as well as the impact of pore shape. Focusing on the water imbibition could not only compress the free gas but also promote the gas desorption as water molecules naturally have superior affinity to solid phase than gas molecules. In 2022, Wang et al. [33] proposed a comprehensive model for nanoconfined water imbibition. In this work, attention is focused on the spontaneous water imbibition at the nanoscale; the other factors associated with water imbibition in shale or coal where gas desorption or compression may take place are excluded here, such as the mentioned displacement pressure, osmotic pressure, and so on. The key parameters for the collected MD simulation results and important features of the models are summarized in Table 1. Additionally, inferred from the brief review, the current models fail to incorporate the spatially varying viscosity at the nanoscale, and the slippage behavior is considered with a fixed slip length that lacks a necessary theoretical basis. Moreover, current models fail to capture the water–surface interaction strength, represented by surface contact angle in this paper, on spontaneous water imbibition behavior at the nanoscale, which may lead to inaccurate results.

**Figure 3:**
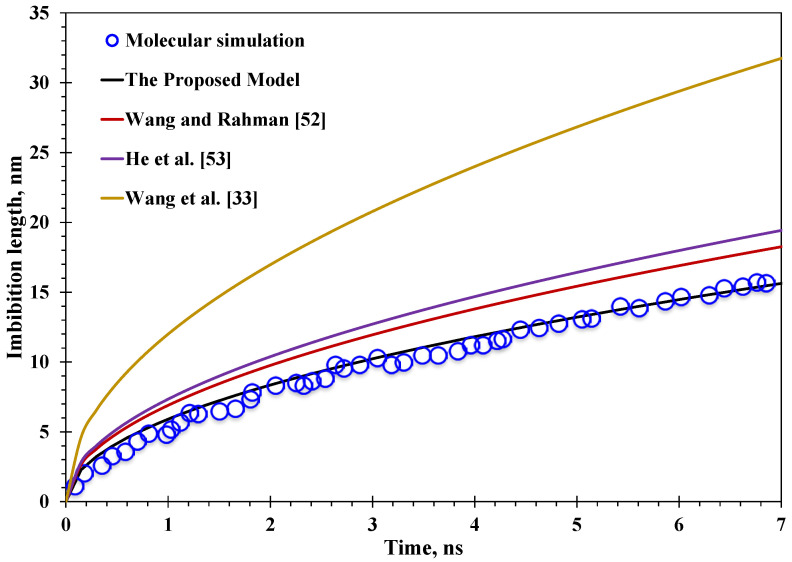
Reliability clarification of the proposed model against existing models and MD simulation data.

As illustrated in Figure 3, all the current models overestimate the spontaneous water imbibition distance at the nanoscale with different magnitudes, in the decreasing order of Wang et al. [33], He et al. [53], and Wang and Rahman [52]. In contrast, the imbibition distance predicted by the proposed model can have a favorable match with the MD simulation results, with an average difference of 2.8%, which is far lower than the recently developed models with overestimation ranging from 16.7% to 103.2%. Inherently, the mechanism behind the overestimation of all the current models is the neglect of the spatially varying water viscosity, which would increase the effective water viscosity and lead to a reduction in water imbibition distance. At the same time, comparing the model of Wang and Rahman [52], with He et al. [53], or Wang et al. [33], the only difference lies in the model of Wang and Rahman [52] neglecting the slippage, which could have improved the nanoconfined water imbibition performance. Considering the slippage behavior but neglecting the nanoconfined enlarging water viscosity would increase the overestimation magnitude of the analytical model. As a result, the overestimation and the relevant different magnitudes are mainly caused by spatially varying water viscosity and wettability-related slip length, well demonstrating the necessity and novelty of this work coupling both factors.

## 5. Results and Discussion

An in-depth investigation is performed in this section to reveal the dependence of nanoconfined water imbibition distance on the wettability effect and its variation with pore size. In addition, the nanopore geometry, including capillary and slit nanopores, could greatly affect the water hydrodynamics and further the spontaneous imbibition behavior, the impact of which is also investigated in this section.

### 5.1. Wettability Effect

**Figure 4:**
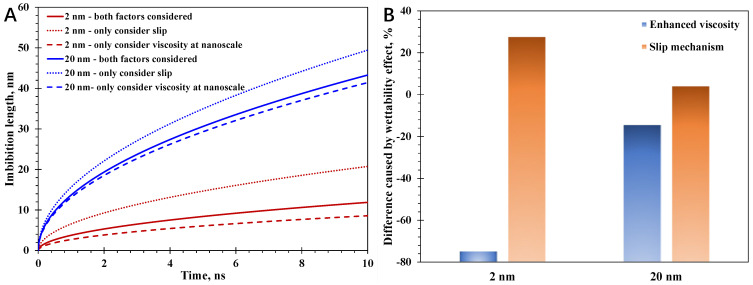
Spontaneous water imbibition distance with different wettability-induced mechanisms considered: (**A**) spontaneous imbibition distance; (**B**) impacts of enhanced viscosity and water slip mechanism.

At first, two pore sizes, 2 nm and 20 nm, are utilized to shed light on the impact of the wettability effect over different pore sizes. Moreover, the nanopore shape is cylindrical with a surface contact angle of 25° in this calculation case. In this work, the wettability effect is classified as the impact of spatially varying water viscosity and slip mechanism. Then, research is performed to identify the impact contributed by different wettability-induced mechanisms. As depicted in Figure 4, it can be observed that the water imbibition distance with a pore size of 20 nm is much greater than that of 2 nm, which can be mainly attributed to the greater viscous resistance in nanoconfined water flow in smaller pores. Meanwhile, regardless of the pore size, the slip mechanism has a positive role in promoting the water imbibition distance, while the enhanced viscosity at the nanoscale reduces the imbibition distance as a negative impact. As for the physical process of spontaneous water imbibition, the effective water viscosity at the nanoscale is greater than the bulk value, since the hydrophilic nanopore surface would lead to a rise in water viscosity at the interfacial area. Therefore, the spatially varying water viscosity always plays a detrimental role in reducing the water flowability at the nanoscale. More importantly, the affecting magnitude of each mechanism on water imbibition varies over different pore sizes. The slip mechanism improves the water imbibition distance inside a 2 nm nanoscale capillary by 28.1%, while the magnitude becomes 6.2% with a pore size of 20 nm. The absolute magnitude caused by enhanced viscosity can affect the water imbibition performance much more than the slip mechanism, reaching as high as 78.6% with a 2 nm pore size, about 3 times that of the slip mechanism. Similarly, the magnitude caused by enhanced viscosity declines with increasing pore size, which is only 19.3% with a 20 nm pore size. Hence, the nanoconfinement impact, indicating both the enhanced water viscosity and slip mechanism, declines tremendously with the increase in pore size. Therefore, it is critical to incorporate the nanoconfinement impact at the nanoscale, particularly for pore sizes less than 20 nm; however, it approaches little impact rapidly and can be overlooked for a large pore size. The observations are consistent with the variation relationship between the wettability-induced mechanisms and pore size: the effective water viscosity will approach the bulk value inside a large pore size, presented by Equation (2), as the volumetric ratio of the interfacial area is close to a minimum. Also, the impact of water slippage is fairly limited within a large pore size, which can be demonstrated by Equation (16).

In order to highlight the varying impacts of different wettability-induced mechanisms over pore sizes, Equations (21) and (22) are proposed, which present the impact by spatially varying water viscosity and slip mechanisms versus pore sizes, respectively. According to the basic formula structure, the value of Equation (21) approaching unity suggests the little impact of enhanced water viscosity at the nanoscale, and the value of Equation (22) approaching zero indicates that the slippage mechanism can be neglected.

(21) $$Ratio_{\mu }=\frac{\mu_{eff}}{\mu_{b}}$$

(22) $$Ratio_{slip}=\frac{l_{s,i}}{R}$$

**Figure 5:**
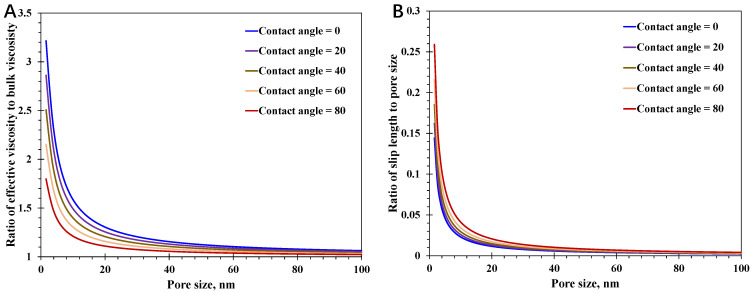
Impact of surface wettability varies dramatically with increasing pore size: (**A**) enhanced water viscosity; (**B**) water slip mechanism.

From Figure 5, it can be observed that the impact of the enhanced viscosity or slippage mechanism declines fast with increasing pore size, both of which can be neglected when pore size exceeds 100 nm. In detail, the dramatic decline takes place with pore size less than 20 nm, and the decline speed for the impact of slippage is faster than the enhanced water viscosity at the nanoscale. In addition, at a certain pore size presented in Figure 5A, the impact of the enhanced viscosity at the nanoscale increases with the decline in surface contact angle, representing the stronger hydrophilic surface. Conversely, as illustrated in Figure 5B, the slippage phenomenon would be greatly mitigated with strong hydrophilic surfaces. Accordingly, with increasing surface contact angle, the impact of the slippage mechanism enlarges while the impact of spatially varying viscosity reduces. In light of the fact that the spontaneous imbibition of nanoconfined water behavior is controlled by both mechanisms, which have completely different variation relationships with the surface contact angle, it is crucial to reveal the influence of surface wettability on water imbibition distance at the nanoscale.

**Figure 6:**
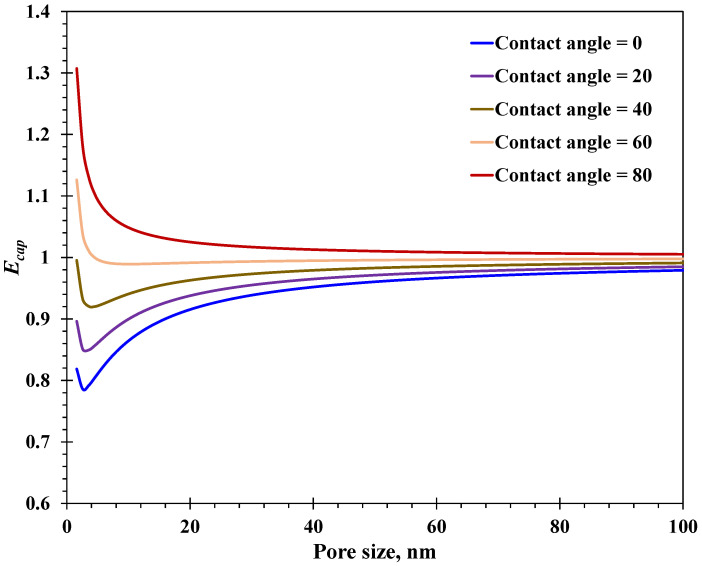
Enhancement factor of nano-capillaries with different surface–water contact angles.

Equation (23) for the enhancement factor is proposed to quantify the difference between the water imbibition distance with wettability impact incorporated and that evaluated by the classic L-W equation. As depicted in Figure 6, the enhancement factor with different surface wettability values has similar variation features with increasing pore size, which declines first and then approaches unity gradually. Notably, for surface contact angles less than 40°, the enhancement factor is less than 1, indicating that the negative impact of enhanced water viscosity is greater than the positive impact of the slippage mechanism. In contrast, when the contact angle exceeds 60°, the contribution of the slippage mechanism outperforms that of the enhanced water viscosity. The first decline in the enhancement factor with increasing pore size is mainly caused by the slippage mechanism, the magnitude of which drops drastically with increasing pore size. After that, the variation trend for the enhancement factor approaching unity is dominated by the spatially varying water viscosity at the nanoscale. In general, at a certain pore size, the water imbibition distance with weak hydrophilic surfaces is greater than that with strong hydrophilic surfaces, reaching as much as 1.3 times the distance predicted by the L-W equation. Also, the strong hydrophilic nanopore surface could lead to an over 20% reduction in the spontaneous water imbibition distance. In short, it is urgent to consider the wettability effect on water imbibition at the nanoscale, which may result in −20–30% deviation compared with the accurate water imbibition behavior.

(23) $$E_{cap}=\frac{L_{cap}}{L_{cap,ori}}$$

### 5.2. Pore Geometry

**Figure 7:**
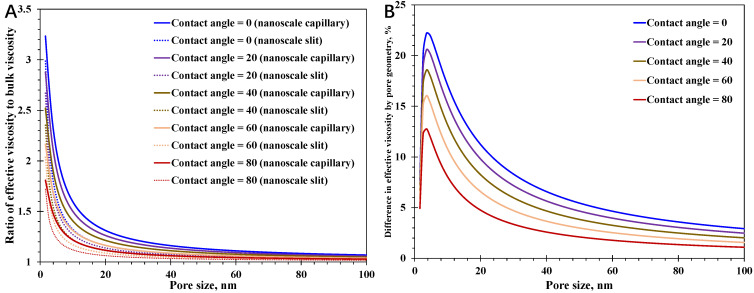
Impact of pore shape on enhanced water viscosity at the nanoscale: (**A**) ratio of effective viscosity to the bulk viscosity; (**B**) difference in nanoconfined viscosity caused by pore shape.

Based on the theoretical analysis, the slippage mechanism is assumed to have the same impact on spontaneous water imbibition regardless of the varying pore geometry, since the key parameter, slip length, is only related to surface contact angle as presented by Equation (13). In comparison, the effective water viscosity at the nanoscale relies heavily on the nanopore geometry, which could have a greater impact on water imbibition behavior than the slippage mechanism. In this regard, attention is mainly put on the nanoconfined spatially varying viscosity here. In Figure 7, the impact of pore geometry on the nanoconfined effective water viscosity with different surface contact angles is presented. At a certain pore size and surface contact angle, the effective viscosity inside the nanoscale capillary is larger than that within the nanoscale slit, indicating that water imbibition in the nano-capillary is harder than in the nano-slit. Meanwhile, with a certain surface contact angle, it can be observed that the effective water viscosity in the nano-slit declines much faster with increasing pore size than in the nano-capillary, suggesting that the difference in the water imbibition performance caused by pore geometry could enlarge first with increasing pore size. The varying features in Figure 7B, characterizing the differences in effective water viscosity by pore geometry, demonstrate the non-linear correlation between the difference and increasing pore size. As for a certain surface contact angle, with increasing pore size, the difference in effective water viscosity caused by pore geometry rises first and then declines gradually, leading to a maximum difference when pore size is close to 5 nm. Also, the maximum value of the difference has a positive relationship with reducing surface contact angle, reaching as high as 22.1%, showing that the nanopore hydrophilic surface enhances the viscosity difference by pore geometry. Moreover, with a relatively large pore size, the difference declines gradually when the effective water viscosity in nanopores with different pore shapes is close to the bulk water viscosity, which is less than 5% when pore size reaches 100 nm.

**Figure 8:**
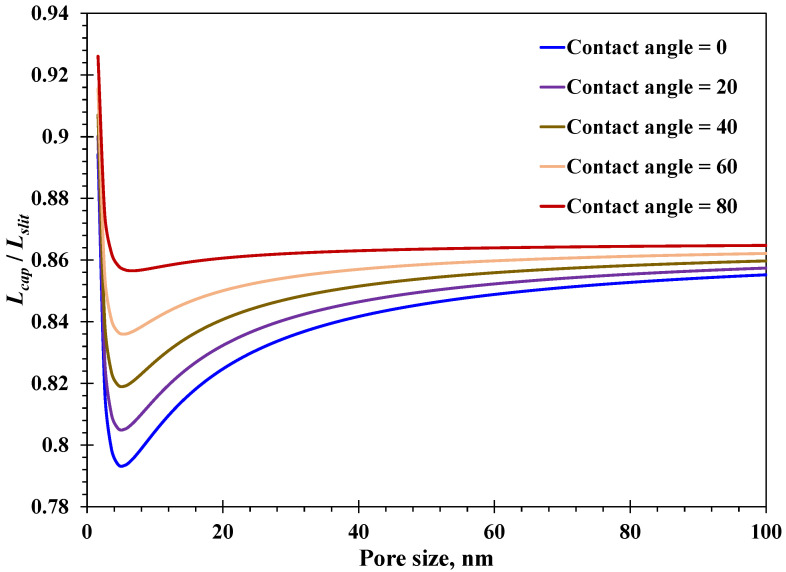
Spontaneous imbibition distance of nanoconfined water with different pore shapes.

Then, the imbibition distance resulting from different nanopore geometries was investigated. From Figure 8, the ratio of water imbibition distance in the nano-capillary to that in the nano-slit versus pore sizes is presented. Regardless of the surface contact angle, the ratio is always less than unity, indicating that the water imbibition performance in the nano-slit is superior to that in the nano-capillary. Also, with a certain surface contact angle, the ratio has a similar variation tendency with increasing pore size. The ratio declines first when pore size is less than 5 nm, and then approaches a stable value around 0.85 with pore size greater than 80 nm. Inherently, as for a relatively small pore size, the wettability effect, encompassing the spatially varying water viscosity and slippage mechanism, would become negligible. Therefore, the mentioned stable value of the ratio around 0.85 is mainly determined by the difference in the hydrodynamics due to different nanopore geometries. As for a small nanopore size where the wettability effect cannot be overlooked, the ratio declines with increasing surface contact angle, which can be attributed to the positive contribution from the slippage mechanism as the weak hydrophilic surface promotes an increase in slip length. The rapid decline in the ratio with pore size around 5 nm occurs for the following two reasons. At first, the impact of the slippage mechanism decreases quickly with increasing pore size, as confirmed in Figure 5B, impairing the spontaneous water imbibition ability. More importantly, as illustrated in Figure 7B, the difference in the nanoconfined effective water viscosity reaches a peak at a pore size of around 5 nm, directly leading to the rapid decline in the ratio in Figure 8. In detail, the minimum ratio could reach as low as 0.79 with extremely hydrophilic nanopore surfaces. Accordingly, the spontaneous water imbibition behavior at the nanoscale is affected by not only hydrodynamics due to the nanopore geometry but also the wettability effect, particularly within pore sizes less than 20 nm. The water imbibition performance in the nano-capillary could be close to that in the nano-slit within a weakly hydrophilic nanopore with pore sizes less than 5 nm, which is a typical case where the wettability effect has a pronounced role in affecting the nanoconfined imbibition behavior. It is critical to consider the wettability effect to accurately predict the nanoconfined water’s imbibition dynamics.

## 6. Conclusions

1. A novel model for spontaneous water imbibition at the nanoscale, incorporating the spatially varying water viscosity and slippage mechanism, both of which are related to surface wettability and keep the critical theoretical consistency, is proposed for the first time. Comparing it with MD simulations clarifies the model’s reliability well, with an average difference of 2.8%, and indicates that the majority of current analytical models overestimate the water imbibition performance at the nanoscale due to the neglect of the enhanced water viscosity under nanoconfinement.
2. The reduction in nanoconfined spontaneous water imbibition distance with enhanced water viscosity at the nanoscale results in could reach as high as 78.6%; conversely, the slippage mechanism promotes imbibition behavior. With increasing pore size, the impact of the slippage mechanism declines faster than the varying water viscosity at the nanoscale, leading to the enhancement factor reducing first and then approaching unity. The wettability effect could result in −20–30% deviation compared with the water imbibition behavior assessed by the classic L-W equation, particularly for pore sizes less than 20 nm.
3. Effective viscosity in the nano-cylinder is always greater than in the nano-slit at a certain contact angle and pore size, leading to the imbibition ability in the nano-slit outperforming that in the nano-capillary. With increasing pore size, the ratio of imbibition distance inside the nano-capillary to nano-slit declines first and then approaches a stable value of 0.85, which is mainly attributed to the non-linear correlation between the difference in nanoconfined viscosity by pore geometry and pore size. The strong hydrophilic surface contributes to the decline in the ratio, the minimum of which reaches as low as 0.79.

## Figures and Tables

**Table 1:** Critical parameters for examining model reliability.

Scholars	Approach	Pore Shape/Size	Slip Length	Spatially Varying Viscosity
Stroberg et al., 2012 [51]	MD simulation	Cylinder, 5 nm	Considered	Considered
Wang and Rahman, 2016 [52]	Analytical model	Cylinder, 5 nm	Not considered	Not considered
He et al., 2017 [53]	Analytical model	Cylinder, 5 nm	A fixed value	Not considered
Wang et al., 2022 [33]	Analytical model	Cylinder, 5 nm	A fixed value	Not considered
The proposed model	Analytical model	Cylinder, 5 nm	Considered	Considered

## Data Availability

The original contributions presented in this study are included in the article. Further inquiries can be directed to the corresponding authors.
